# Effect of early clinical exposure based health systems science course in the Korean medical education: a prospective observational study

**DOI:** 10.1186/s12909-025-08250-z

**Published:** 2025-12-26

**Authors:** Hye-Yoon Lee, Sunju Im, Sang-won Shin, Jeong-Ae Kim, Jin-Mi Song

**Affiliations:** 1 Division of Humanities and Social Medicine, Pusan National University School of Korean Medicine, Yangsan, Republic of Korea; 2 Department of Medical Education, Pusan National University School of Medicine, Yangsan, Republic of Korea; 3 Pusan National University School of Korean Medicine, Yangsan, Republic of Korea

**Keywords:** Health systems science, Early clinical exposure, Korean medicine, Medical humanities and social medicine, Systems thinking, Systems-based practice, Medical education

## Abstract

**Background:**

Health professions are required to understand social structures and systems, as well as basic and clinical medical knowledge. Accordingly, Health Systems Science (HSS) is recognized as a third party of medical education. The curriculum for HSS is currently being developed and implemented in Korea. This study aimed to evaluate the effectiveness of HSS in Korean medical education.

**Methods:**

The HSS course using early clinical exposure (ECE) was implemented in Korean medical education and comprised lectures, four ECEs, and group activities. Students and clinical directors evaluated the course using questionnaires focused on systems thinking, productivity of group activities, and satisfaction.

**Results:**

The study showed significant improvements in systems thinking, especially in team learning (*p* = .028) and overall systems thinking (*p* = .017). Key habits related to systems thinking also improved significantly over 15 weeks. ECE reports highlighted student engagement with various HSS domains, focusing on patient care, healthcare structure, and policy. Per the clinical directors’ evaluations, “healthcare structure and process” was the most effective domain among the core domains of HSS. Per the students’ evaluations, the overall satisfaction was higher than the college’s average score, and especially “course structure” and “instructional expertise” domains were rated most effective.

**Conclusion:**

The HSS course with ECE is an effective method for fostering students’ systems thinking habits and enhancing their understanding of healthcare systems and patient-centered care. Verifying whether students’ interest in various domains and their systems thinking abilities are sustained over time is necessary, and further research is required to design a longitudinal course with continuity.

**Supplementary Information:**

The online version contains supplementary material available at 10.1186/s12909-025-08250-z.

## Background

 The medical education curriculum comprises basic and clinical medicine as well as disciplines in medical humanities and social medicine disciplines [1]. Medical humanities and social medicine gained prominence later than basic and clinical medicine, prompted by the recognized limitations of the Flexner’s model and the call for a more holistic approach to medical education [2, 3]. These fields encompass diverse disciplines such as history, philosophy, ethics, law, religion, and social policy. More recently, Health Systems Science (HSS) has emerged within the domains of medical humanities and social medicine, focusing specifically on healthcare system [4, 5]. HSS provides a comprehensive understanding of the interrelated factors that influence patient health, with the ultimate goal of providing patient-centered medicine. Compared to traditional medical humanities and social medicine, HSS is regarded as more practical and closely linked to real-world healthcare delivery. Recent empirical studies demonstrate that HSS more effectively bridges basic and clinical sciences by addressing complex system-level interactions in health care [6, 7]. In addition, systems thinking is a key element in HSS which refers to the capacity to perceive healthcare not as isolated events but as a dynamic and interconnected system. Systems thinking functions as a thread that integrates multiple HSS domains and is considered an essential competency for future physicians. To evaluate the effectiveness of HSS education, it is therefore important to assess learners’ systems thinking [8]. In this study, we combined two complementary measures: the widely used *Habits of a Systems Thinker* framework, adapted into a Likert scale, and a Korean-developed scale of systems thinking that has demonstrated psychometric validity [9].

HSS addresses the impact of various factors, including healthcare policy, economics, and structure, on patient health status and healthcare experiences. The dualized healthcare system in Korea, where conventional and Korean medicine (KM) coexist, is unique because it offers patients more options. However, it has been criticized for escalating medical costs and causing conflicts among medical professionals [10, 11]. This healthcare system requires professionals’ attention to social aspects beyond clinical practice. HSS is considered more suitable for the four-year medical school program because its scope is narrower and more focused. In contrast, the ‘humanities and social medicine’ concept is deemed more appropriate for the six-year medical college curriculum because it encompasses broader areas, including liberal arts [12]. South Korea has four- and six-year educational programs. The six-year program, which comprised two-year pre-medical and four-year medical courses, has been reformed into a single six-year program. In addition, the South Korean university curricula are categorized into major-required courses and general education courses, with students demonstrating significantly different attitudes towards mandatory courses than general education courses. Considering these factors, it is essential to evaluate which is more suitable between HSS and the humanities and social medicine framework, or whether combining both should be considered. In addition, students tend to show low interest in humanities and social medicine and regard these subjects as unrelated to their primary studies [13]. Therefore, it is essential to identify appropriate teaching methods for HSS.

Early clinical exposure (ECE) refers to exposure to clinical settings in hospitals and the community before the clinical phase, helping bridge the gap between theory and practice [14]. ECE addresses limitations of traditional lecture-based instruction such as one-way knowledge transmission, rote memorization, and exam-focused assessment, by promoting experiential and self-directed learning. Frequent exposure to clinical practice not only enhances understanding of medical fields but also fosters empathy for patients’ suffering and motivation for professional growth [15].

In terms of Korean Medicine, most colleges of Korean Medicine adopt a 6-year undergraduate curriculum consisting of a 2-year premedical course and a 4-year medical course, leading to eligibility for the national licensing examination. One institution has adopted a graduate-entry “professional school” model, admitting students who already hold a bachelor’s degree and providing a 4-year curriculum toward the same licensure. This school also offers a 7-year combined bachelor’s/master’s program, structured as three years of bachelor-level study followed by four years of the graduate medical curriculum. All three tracks—6-year undergraduate, 4-year graduate, and 7-year integrated—qualify graduates to sit for the Korean Medicine licensing examination. At a time when the introduction of HSS curricula is being actively discussed, this study aimed to evaluate an HSS course implemented in the bachelor-level phase of a 7-year integrated bachelor’s/master’s program. In this study, we aimed to introduce HSS into Korean medical education and evaluate its satisfaction and effectiveness.

## Methods

### Study design

This prospective observational study collected students’ responses to questionnaires on the study’s purpose and analyzed the outcomes of the course, including the reaction and learning. The Pusan National University Institutional Review Board approved this study (No.2023-150-HR).

### Subjects

All third-year undergraduate students (*n* = 21) in the combined Bachelor-Master program in Korean Medicine (3 + 4) program undertook the course in the second semester of 2023. The students studied basic biomedical sciences and basic liberal arts before taking any clinical course.

### Consent and recruitment for the questionnaires

The questionnaires were administered to only participants who submitted written informed consent. Information regarding study participation was posted on a website and social media. Interested students were provided with written explanations. They were given opportunities to ask questions, after which the students who voluntarily agreed to participate signed a written consent form. The surveys were anonymous and collected no personal information. Participants tracked changes using self-chosen nicknames.

### Teaching methods (activities during this course)

#### Lectures

**Fig. 1:**
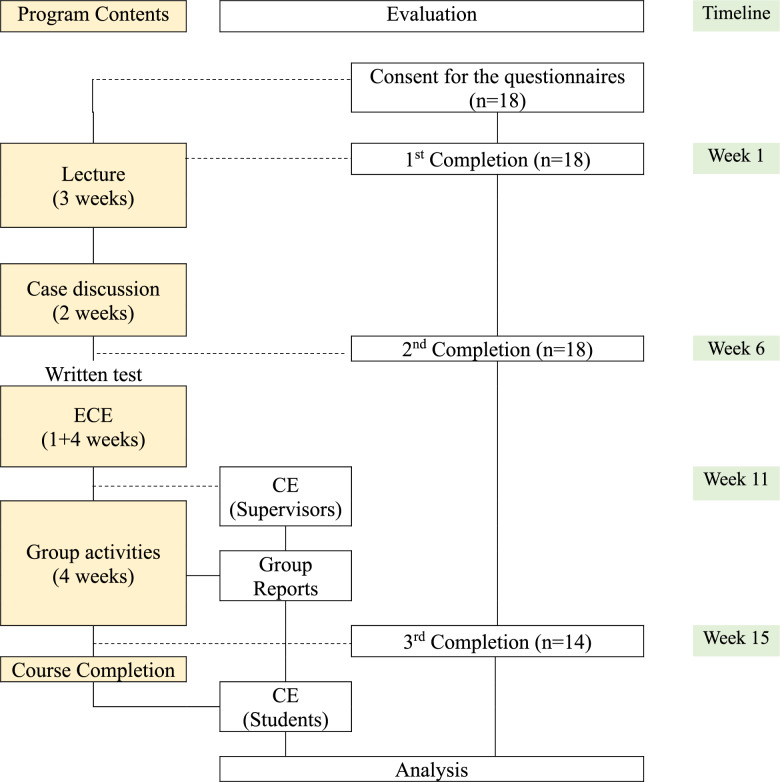
The flow diagram of the course and the evaluation. ECE, early clinical exposure (1week for preparations and 4weeks in the field); CE, course evaluation. ‘Questionnaires’ includes ‘Group activities’ indicates the basic and advanced group activities. ‘Group reports’ indicates the early clinical exposure and in-depth investigation reports

The lectures explained the concept of HSS core functional, foundational, and linking domains, highlighting that HSS is the third pillar of medical education in basic and clinical sciences. A total of 3 lecture sessions (6 h) were delivered by three professors: (1) a medical doctor and a medical education expert, (2) a Korean medical doctor (KMD) and expert in classics in Korean medicine, and (3) a KMD (specialist in Korean internal medicine) and expert in medical education (Fig. 1).

#### Case-based discussion

Two separate sessions (4 h) were dedicated specifically to in-depth case-based discussions.

The professors developed cases illustrating the complex interrelationships between various domains within HSS. During class, the students analyzed the underlying HSS domains of each case and systematically approached solutions using the think-pair-share method. Below are the case examples used in the discussion:

- “Let us consider the causes and solutions for a 46-year-old male patient with obesity.”
- “Let us consider the causes and prevention methods of incidents where acupuncture needles are unintentionally left in a patient’s body after treatment at a KM clinic.”

#### Early clinical exposure

We formed 10 groups comprising nine duos and one trio and assigned each group to a KM clinic based on their preferences. Over four consecutive weeks, the students visited their designated clinic once weekly for 3 h over. During their visits, the students interviewed the patients, focusing on the interconnections between HSS domains.

#### Basic group activity – ‘early clinical exposure report’

After completing 4-weeks of ECE training, the students systematically analyzed their observations according to HSS domains. Then, each group presented their findings, followed by a Q&A session.

#### Advanced group activity – ‘in-depth investigation report’

During the ECE, each group was free to choose, research, and present a topic of interest.

### Course evaluation methods

#### The scale of systems thinking

The scale of Systems Thinking, a previously developed questionnaire [9], is embedded in the Pusan National University Learning Management System (LMS). The questionnaire was anonymous, and the students used nicknames to track changes over time (Weeks 1, 6, and 15). The scale comprised 20 items on a 5-point Likert scale categorized into five sub-factors: mental model, individual skill, team activity, systems thinking, and shared vision. (Supplementary file 1)

#### Self-assessment of the habits of a systems thinker

We explained the 14 Habits of a Systems Thinker presented by the Waters Center for Systems Thinking to the participants [16]. We asked the students to rate their competence on a 5-point Likert scale for each item. This questionnaire was embedded in the Pusan National University LMS, and a similar method was used to maintain anonymity and track changes.

The 14 habits are as follows: “H1, Seeks to understand the big picture; H2, observes how elements within systems change over time, generating patterns and trends; H3, recognizes that a system’s structure generates its behavior; H4, identifies the circular nature of complex cause-and-effect relationships; H5, make a meaningful connection within and between systems; H6, Changes perspectives to increase understanding; H7, surfaces and tests assumptions; H8, considers an issue fully and resists the urge to come to a quick conclusion; H9, considers how mental models affect current reality and the future; H10, uses understanding of system structure to identify possible leverage actions; H11, considers short-term, long-term and unintended consequences of actions; H12, pays attention to accumulations and their rates of changes; H13, recognizes the impact of time delays when exploring cause and effect relationships; H14, checks results, and changes actions if needed: successive approximation” [16]. (Supplementary file 2)

#### Early clinical exposure report

We analyzed reports systematically summarizing observations made during ECE at each KM clinic according to HSS core domains. The researchers extracted meaningful insights from 10 reports and categorized them according to HSS domains using thematic analysis [17, 18].

#### In-depth investigation report

Reports containing detailed investigations on topics of group interest were analyzed and presented. Meaningful insights, which were defined as reflections that went beyond simple observation but contain understanding of HSS domains or their relationship, were extracted. They are categorized according to AMA HSS domains framework, which serve as the analytical lens for categorizing the emerging themes [17, 18].

#### Course evaluation survey of the clinical supervisors

After completing the students’ field study, a survey was administered to the supervising KMD to assess the program’s effectiveness. This survey was structured according to the AMA HSS framework, comprising three broad domains: core functional, foundational, and linking. Within these, the core functional domain included seven subdomains, the foundational domain consisted of four subdomains, and the linking domain was represented by one subdomain of systems thinking. Supervisors rated each item on a 5-point Likert scale (1 = very poor/strongly disagree, 2 = poor/disagree, 3 = adequate/neutral, 4 = good/agree, 5 = excellent/strongly agree).

#### Course evaluation by students

**Fig. 2:**
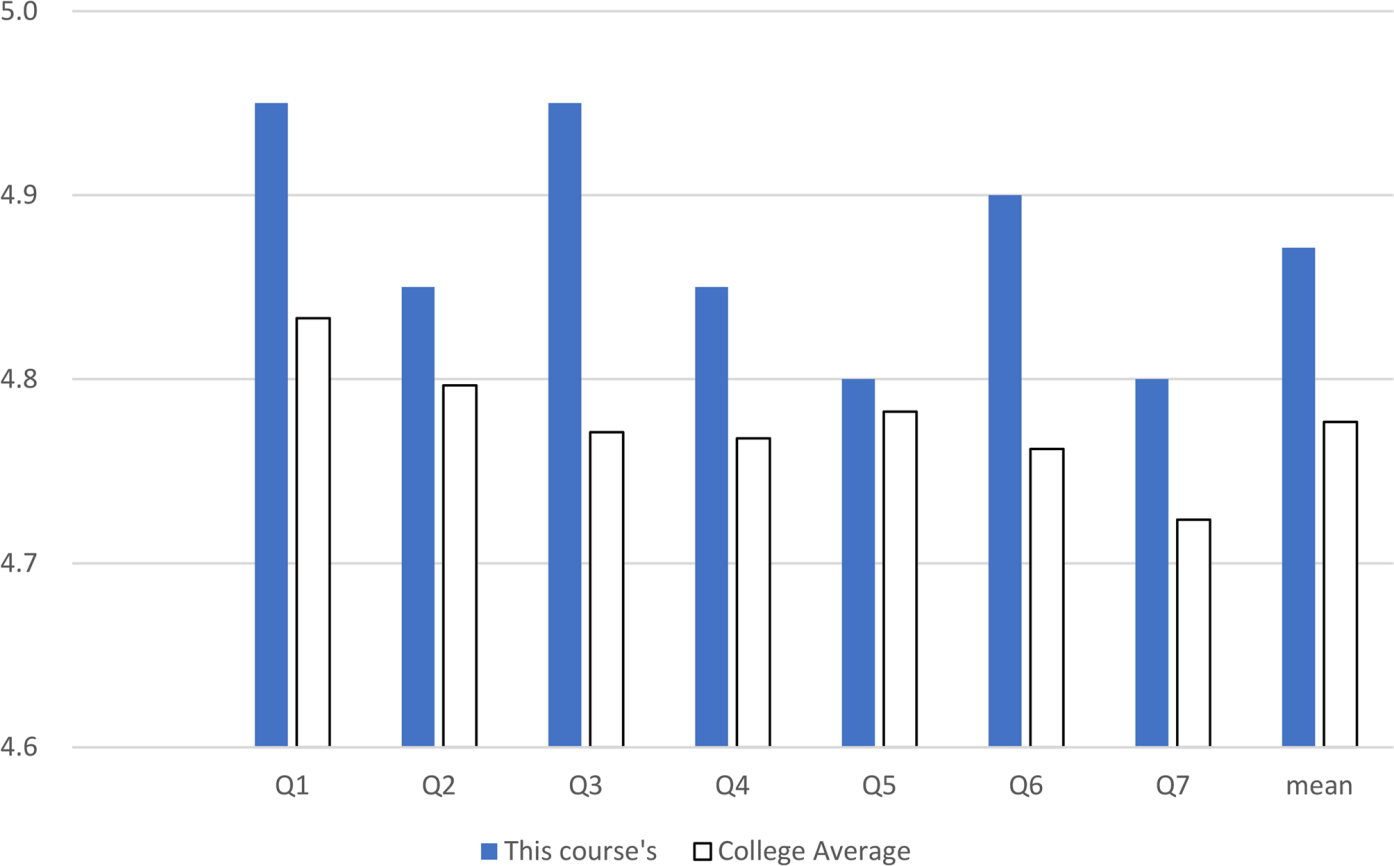
The end-term course evaluation results by students. For better readability, the graph starts from a score of 4.5. Q1: Course Structure (syllabus); Q2, Teaching Methods; Q3, Instructional Expertise; Q4, Assignments (feedback); Q5, Evaluation Criteria; Q6, Effectiveness; Q7, Satisfaction

The end-of-term evaluation was conducted using the official evaluation form routinely administered by the university at the conclusion of all courses. This instrument comprised seven items assessing course structure (syllabus), teaching methods, instructional expertise, assignments (including feedback), evaluation criteria, effectiveness, and overall satisfaction. Students rated each item on a 5-point Likert scale (1 = very poor/strongly disagree, 2 = poor/disagree, 3 = adequate/neutral, 4 = good/agree, 5 = excellent/strongly agree). The mean scores of these evaluations were utilized as outcome measures in this study. The evaluation results for this course were compared with average college scores (Fig. 2).

### Statistical analysis

The survey results were statistically analyzed using descriptive and inferential statistics to evaluate the effects before and after the educational intervention. Descriptive statistics are presented as means and standard deviations. Due to the small sample size, pre- and post-comparisons were performed using non-parametric tests, specifically the Wilcoxon signed-rank test. All statistical analyses were performed using the IBM statistical package for social sciences version 29. Statistical significance was defined as a two-tailed *p*-value < 0.05.

## Results

All students completed the course and submitted an end-term course evaluation. Of the students, 18 of 21 (85.7%) submitted written consent forms for the questionnaires, and 14 of the 18 participants (77.8%) completed the three times questionnaires (Fig. 1).

### The scale of systems thinking

This tool assesses various dimensions of systems thinking, including mental models, personal mastery, team learning, shared vision, and systems thinking.

The Scale of Systems Thinking assessed five domains: mental models, personal mastery, team learning, shared vision, and systems thinking. In the Mental Model category, mean scores remained stable from week 1 (3.8 ± 0.6) to week 6 (3.8 ± 0.5), but increased significantly at week 15 (4.0 ± 0.5), *p* =.041. Team Learning improved significantly from week 1 (3.4 ± 0.6) and week 6 (3.4 ± 0.6) to week 15 (3.8 ± 0.8), *p* =.028. The Systems Thinking category also showed significant enhancement, increasing from week 1 (3.6 ± 0.5) to week 6 (3.7 ± 0.5), *p* =.041, and further to week 15 (4.2 ± 0.5), *p* =.017. The total score rose significantly from week 1 (3.8 ± 0.4) and week 6 (3.8 ± 0.5) to week 15 (4.1 ± 0.4), *p* =.021, highlighting an overall enhancement in systems thinking abilities throughout the study (Table 1).

**Table 1:** The scale of systems thinking (*n*=14, mean (SD))

	Week 1	Week 6	Week 15	Z^a^	*p*-value^a^	Z^b^	*p*-value^b^	Z^c^	*p*-value^c^
Systems Thinking	3.6 (0.5)	3.7 (0.5)	4.2 (0.5)	-.464	.642	-2.422	.015*	-2.395	.017*
Mental Model	3.8 (0.6)	3.8 (0.5)	4 (0.5)	-.179	.858	-2.040	.041*	-1.395	.163
Personal Mastery	4.1 (0.4)	4 (0.6)	4.2 (0.4)	-1.132	.258	-1.568	.117	-.270	.787
Shared vision	3.9 (0.8)	3.9 (0.7)	4.2 (0.6)	-.419	.675	-1.490	.136	-1.163	.245
Team Learning	3.4 (0.6)	3.4 (0.6)	3.8 (0.8)	-.275	.784	-1.587	.112	-2.195	.028*
Total (average)	3.8 (0.4)	3.8 (0.5)	4.1 (0.4)	-.912	.362	-2.043	.041*	-2.310	.021*

*p*-value derived by Wilcoxon Signed-Rank test

^a^Changes between weeks 6 and 1

^b^ Changes between weeks 15 and 6

^c^ Changes between weeks 15 and 1

*p-value <0.05

In contrast, Personal Mastery showed minor fluctuations across week 1 (4.1 ± 0.4), week 6 (4.0 ± 0.6), and week 15 (4.2 ± 0.4), with no significant changes (*p* >.05). Shared Vision scores also remained consistent (week 1: 3.9 ± 0.8; week 6: 3.9 ± 0.7; week 15: 4.2 ± 0.6), with no significant differences detected (*p* >.05).

### Self-assessment of a systems thinker’s habits

The self-assessment tool measured 14 habits of systems thinking over 15 weeks.

Significant improvements were primarily observed between weeks 6 and 15, as well as over the entire period from week 1 to week 15, rather than in the early phase (weeks 1 to 6). H1 (Big Picture) increased from 3.9 ± 0.8 at week 1 to 4.4 ± 0.7 at week 15 (*p* =.035). H2 (Change Over Time Patterns) showed gains from 3.9 ± 0.7 at week 6 to 4.3 ± 0.5 at week 15 (*p* =.034) and from 3.6 ± 1.0 at week 1 to 4.3 ± 0.5 at week 15 (*p* =.047). H9 (Mental Model) rose from 3.2 ± 0.8 at week 1 to 3.9 ± 0.7 at week 15 (*p* =.026), while H10 (Leverage Points) increased significantly from 3.2 ± 0.9 at week 6 to 4.1 ± 0.7 at week 15 (*p* =.014).

Improvements limited to the later phase (weeks 6–15) were found in H11 (Short/Long/Unintended Consequences), increasing from 3.5 ± 1.1 at week 6 to 4.4 ± 0.6 at week 15 (*p* <.05), H13 (Time Delays in Causality) from 3.3 ± 0.7 to 4.3 ± 0.5 (*p* <.01), and H14 (Check & Adjust Actions), rising from 4.0 ± 0.7 to 4.6 ± 0.6 (*p* <.05).

Other habits, such as H3 (Structure Drives Behavior) and H6 (Perspective Shifts), showed positive but non-significant trends throughout the period.

The overall mean score improved significantly from 3.6 ± 0.5 at week 1 to 4.2 ± 0.5 at week 15 (*p* =.002), indicating an overall enhancement in systems thinking habits (Table 2).

**Table 2:** Self-assessment of a systems thinker’s habits (*n*=14, mean (SD))

Habits	Week 1	Week 6	Week 15	Z^a^	*p*-value^a^	Z^b^	*p*-value^b^	Z^c^	*p*-value^c^
H1. Big picture	3.9 (0.8)	3.8 (0.9)	4.4 (0.7)	-.302	.763	-1.653	.098	-2.111	.035*
H2. Change over time patterns	3.6 (1)	3.9 (0.7)	4.3 (0.5)	-.966	.334	-2.121	.034*	-1.983	.047*
H3. Structure drives behavior	3.6 (0.9)	3.8 (0.6)	4.0 (0.7)	-.632	.527	-1.342	.180	-1.890	.059
H4. Circular cause-effect	3.6 (0.9)	3.8 (0.8)	3.9 (0.9)	-.707	.480	-.447	.655	-1.000	.317
H5. Connections within/between systems	3.6 (1)	3.7 (1)	4.1 (0.9)	-.412	.680	-1.249	.212	-1.408	.159
H6. Perspective shifts	3.7 (1)	3.8 (1)	4.1 (0.7)	-.108	.914	-1.406	.160	-1.857	.063
H7. Test assumptions	3.4 (1.1)	3.6 (1)	3.9 (0.9)	-1.190	.234	-1.100	.271	-1.613	.107
H8. Thorough issue consideration	3.8 (0.9)	3.9 (0.9)	4.4 (0.8)	-.378	.706	-1.811	.070	-1.565	.118
H9. Mental model	3.2 (0.8)	3.6 (0.8)	3.9 (0.7)	-1.406	.160	-1.667	.096	-2.233	.026*
H10. Leverage points	3.4 (0.9)	3.2 (0.9)	4.1 (0.7)	-.586	.558	-3.326	.020*	-2.460	.014*
H11. Short/long/unintended consequences	3.5 (1)	3.5 (1.1)	4.4 (0.6)	-.054	.957	-2.209	.027*	-2.588	.010*
H12. Accumulation & change rates	3.8 (0.8)	3.6 (0.5)	4.1 (0.8)	-.632	.527	-1.539	.124	-1.890	.059
H13. Time delays in causality	3.2 (0.9)	3.3 (0.7)	4.3 (0.5)	-.333	.739	-3.125	.002*	-2.877	.004*
H14. Check & adjust actions	4.1 (1)	4 (0.7)	4.6 (0.6)	-.345	.730	-1.994	.046*	-2.333	.020*
Total (average)	3.6 (0.5)	3.7 (0.5)	4.2 (0.5)	-.534	.593	-3.111	.002*	-3.061	.002*

*H1* Seeks to understand the big picture, *H2*, observes how elements within systems change over time, generating patterns and trends, *H3* recognizes that a system’s structure generates its behavior, *H4* identifies the circular nature of complex cause-and-effect relationships, *H5* make a meaningful connection within and between systems, *H6* Changes perspectives to increase understanding, *H7* surfaces and tests assumptions; H8, considers an issue fully and resists the urge to come to a quick conclusion, *H9* considers how mental models affect current reality and the future, *H10* uses understanding of system structure to identify possible leverage actions, *H11* considers short-term, long-term and unintended consequences of actions, *H12* pays attention to accumulations and their rates of changes, *H13* recognizes the impact of time delays when exploring cause and effect relationships, *H14* checks results, and changes actions if needed: successive approximation

^a^ Changes between weeks 6 and 1, ^b^ Changes between weeks 15 and 6, ^c^ Changes between weeks 15 and 1

*p*-value derived by Wilcoxon Signed-Rank test; *p-value <0.05

### ECE reports and the HSS core domains

The topics derived from students’ learning activities are ECE and in-depth investigation reports (Table 3). They were categorized according to HSS domains. Any report with multiple topics was divided according to the topics, and similar reports were consolidated into a single entry.

**Table 3:** Health Systems Science core functional domains and students’ learning activities

Domains	Early clinical exposure report	In-depth investigation report
Patients, family, and community	• Visited upon recommendation from an acquaintance. • Daughter came for treatment, previously accompanying father. • Aspiring compassionate doctor who cares for patients in traditional Korean medicine. • Roles patients expect from a Korean medicine clinic (consultation, lifestyle management, and initial herbal treatments).	• Based on lumbar disc herniation patient's occupation, environment, and lifestyle management. • Factors contributing to patient satisfaction • Patients' expectations and traditional knowledge explanations (e.g., pulse diagnosis).
Health care structure and process	• Diagnosis process within the clinic. • Differences between clinic-level and hospital-level medical institutions. • Role and challenges in healthcare delivery systems.	• Differences between Korean medicine clinics and hospitals • Roles and challenges in healthcare delivery systems.
Health care policy and economics	• Elderly fixed payment system, collaborative pilot projects, contract practice pilot projects, and home visit pilot projects. • Automobile insurance, indemnity insurance, and other reimbursement methods.	• Collaboration pilot projects. • Relationship between health insurance and medical use. • Insurance coverage of acupuncture treatments.
Clinical informatics and health technology	• Use of EMR, HIRA electronic claims, research and writing, questionnaires, scales, and clinical guidelines.	• Types, ingredients, effects, and side effects of acupuncture treatments. • Mechanisms of cupping and bloodletting therapies. • Clinical guidelines. • AI chatbot usage for counseling and marketing in Korean medicine clinics. • Custom keyword settings for Korean medicine clinic promotion.
Population, public, and social determinants of health	• Regional characteristics. • Differences in health status based on patient's occupation and socioeconomic status. • Occupational guidance.	• Major treatment subjects depending on Korean medicine clinic location.
Value in health care	• Cost-effectiveness • Activities to improve quality (outcome, safety, and service) • Measures for preventing accidents. • Waste disposal, safe bedding, and prevention of abrasions/falls/infections.	• International standardization of cupping therapy for quality control.
Health system improvement	• Intra/extra system improvement activities. • Attendance at conferences and lifelong learning. • Treatment room structure for privacy protection.	• Management and marketing (AI chatbot usage).

*AI* artificial intelligence, *EMR* Electronic Medical Record, *HIRA* Health Insurance Review & Assessment Service

#### Patient, family, and community

Students observed the impact of patients’ experiences and beliefs on their medical center usage, noting instances such as “a former guardian of a patient visiting the same medical center as a patient” and “patients choosing medical centers based on recommendations from friends or close acquaintances.” Factors contributing to continuous visits to a KM clinic included “patients feeling genuinely cared for and receiving kind treatment.” The students analyzed patients’ recognition of health issues and their expectations for “conventional medicine,” “KM,” and “lifestyle management,” including the interplay between these elements.

#### Health care structure and process

Regarding the in-clinic structure and process, students closely examined the “in-clinic consultation process,” paying attention to the clinic’s structure and procedures for “patient reception,” “waiting,” “consultation,” and “treatment.” Students also observed collaborations among various healthcare professionals.

For the overall healthcare structure and processes, students mainly discussed roles and challenges within the “healthcare delivery system.”

#### Health care policy and economics

The students were informed about various “government policies” and their impact on “healthcare utilization.” Specific policies included the “Elderly Fixed Outpatient Copayment,” “pilot projects of collaborative treatments between Western and Korean medicine,” “pilot project of herbal decoctions coverage in the national health insurance,” and “pilot project of home-based primary care.” They stated that various “payment and compensation systems,” such as “automobile insurance,” “industrial accident compensation insurance,” and “medical aid,” influenced healthcare utilization patterns.

#### Clinical informatics and health technology

The students compared the advantages and disadvantages of “Electronic Medical Records (EMRs)” and “handwritten charts” and learned how to handle EMRs practically. They observed the electronic claims submitted to the Health Insurance Review and Assessment Service.

In addition, they noted how the supervising KMD utilized “research results,” “clinical guidelines,” and “disease-specific scales” in practice and how the doctor published and presented their clinical outcomes in “research papers.”

#### Population, public, and social determinants of health

The students received explanations regarding the characteristics of the population in the area where the clinic was located. They observed differences in “health status” according to patients’ “socioeconomic status” and “occupation,” and they monitored lifestyle guidance tailored to patients’ “occupational and environmental contexts.”

#### Value in healthcare

The students observed measures that KMDs must adhere to within the clinic to prevent “safety incidents,” such as “waste disposal,” systems for “safe needle removal,” and various actions to prevent “cuts,” “falls,” and “infections.” They learned that quality versus cost is critical, and that quality is determined not only by outcomes, but also safety and service.

#### Health system improvement

The students witnessed “continuous learning” examples, such as the clinic director attending “conferences on weekends” and system improvement strategies, such as identifying and improving inconvenient aspects of the clinic’s structure, including measures for “privacy protection” and “accident prevention.” (Supplementary file 3).

### In-depth investigation report

#### Patient, family, and community

The research focused topics included the “Korean medical management of patients with herniated intervertebral disc (HIVD) based on their occupation, environment, and previous medical experiences,” “factors contributing to loyalty to a KM clinic,” and “patients’ expectations of traditional knowledge and methods of explaining it to patients (such as pulse diagnosis).”

#### Health care structure and processes

The students investigated the differences between “KM clinics and hospitals,” including primary patient groups and institutional differences between clinic-level and hospital-level facilities, the number of inpatients allowed, the required number of physicians and KMDs, and differences in medical costs.

#### Health care policy and economics

One group focused on the “collaborative treatment pilot project between Western and Korean medicine,” examining the government-defined concept of ‘collaborative treatment’ and the conditions included in the pilot project. Under the theme “relationship between indemnity insurance and medical utilization,” the students studied the changes in including KM treatments in Korean indemnity insurance over time, addressing patients’ and doctors’ “moral hazard.” They also explored the insurance system for “pharmacopuncture,” learning about the conditions and significance of insurance coverage.

#### Clinical informatics and health technology

The students examined the “mechanism of cupping therapy on acupoints in the back,” investigating the therapeutic mechanisms of cupping therapy and the relationship between acupoints and autonomic ganglia. They studied the " pharmacopuncture components, effects, and side effects.” The students learned about the “standardization of Korean medical treatments” by investigating the development and use of clinical guidelines. In addition, they explored the use of science and technology, such as “artificial intelligence (AI) chatbot consultations” and the setting of “customized keywords.”

#### Population, public, and social determinants of health

The students studied the “location of KM clinics” and their “major treatment subjects.”

#### Value in health care

The students examined a case in which a “Korean device was designated as the international standard for cupping” and learnt about international standards emphasizing “patient safety” and “infection prevention.” They realized that, in terms of quality versus medical cost, patient safety is a crucial consideration. They also recognized that safety issues are related to outcomes and service, and can lead to an overall increase in healthcare costs.

### Course evaluation of the clinical supervisors

The clinical supervisors responded that the ‘healthcare structure and process’ is the most effective domain to educate students under this program (4.6 ± 0.5), followed by ‘patient, family and community (4.5 ± 0.5)’, ‘clinical informatics and health technology (4.1 ± 0.8)’ and ‘value in healthcare (4.0 ± 0.7)’. The domain that scored lowest was ‘health system improvement (3.9 ± 0.7)’.

Leadership was the most effective fundamental domain (4.2 ± 0.9), to educate with this program.

For the linking domain, “systems thinking” scored 4.2 ± 0.9 (Table 4).

**Table 4:** Effectiveness of this course to educate each domain of HSS (by clinical supervisors, *n*=10)

Category	mean	SD	Domains	mean	SD
Core functional domains	4.2	0.6	Patients, family, and community	4.5	0.5
			Health care structure and process	4.6	0.5
			Health care policy and economics	4.0	0.8
			Clinical informatics and health technology	4.1	0.8
			Population, public, and social determinants of health	4.0	0.8
			value in health care	4.1	0.7
			Health system improvement	3.9	0.7
Foundational domains	4.1	0.7	Change agency, management, and advocacy	4.1	0.7
			Ethics and legal	4.1	0.8
			Leadership	4.2	0.9
			Teaming	4.1	0.7
Linking domain	4.2	0.9	Systems thinking	4.2	0.9

### Course evaluation by students

After completing the education and assessment, an end-term course evaluation was conducted. Of the course evaluation survey items, ‘course structure’ and ‘instructional expertise’ scores were highest (4.95 ± 0.2), followed by ‘effectiveness, ‘‘teaching methods,’ ‘assignments and feedback, ‘evaluation criteria,’ and ‘satisfaction.’ The college average had the lowest score. Notably, ‘instructional expertise’ showed the largest gap, while ‘evaluation criteria’ showed the smallest gap.

## Discussion

In this study, the HSS course supplied the basic concept through minimal lectures and primarily implemented ECE and group activities. Since this was the first implementation of HSS at our institution, direct comparisons with prior courses were not available. However, unlike the traditional course structure primarily based on lectures, the introduction of Early Clinical Exposure significantly reduced lecture time and shifted the focus toward experience-based, in-depth learning activities.

After evaluation, this program improved systems thinking competency and engaged students. Significant improvements were demonstrated in students’ systems thinking abilities, notably in team learning (*p* =.028) and overall systems thinking (*p* =.017). Key cognitive habits related to systems thinking also showed significant progress over the 15-week period. In addition, active engagement was highlighted during ECE with diverse HSS domains including health care structure and policy. According to clinical directors’ evaluations, the “healthcare structure and process” domain was identified as the most impactful among the core HSS domains. Student satisfaction with the course exceeded the college average. These findings imply the combination of reduced lectures, hands-on clinical exposure, and subsequent group projects enhanced students’ active engagement and practical understanding, aligning well with the goals of high-yield instructional methods.

Regarding the SST and 14-HST results, the understanding of time flow increased notably in items such as H11 (considering long- and short-term consequences), H13 (time delays in cause–effect), and H14 (recognizing multiple perspectives over time). This improvement maybe attributed to students being asked to visit a clinic at the same time over a four-week period; observing the patients’ follow-up process helped them monitor changes over time. During the final patient interviews which primarily focused on patients with chronic diseases, the patients discussed their journey from the treatment onset to the present. This likely influenced our results [19].

Recognizing that mental models affect the perception of reality was noted in the 14-HST. However, in the SST, only systems thinking and team learning increased, with a lesser increase in mental models, personal mastery, and shared vision. Unlike the 14-HST, which recognizes the importance of mental models, the SST assesses the actual state of mental models. The students were trained to view phenomena systemically (considering the big picture and changes over time), but more time and training were required to change their mindset (mental model). Personal mastery questions inquiring if one consistently behaved in a systems thinking manner indicated that such foundational mindsets and daily life skills were insufficiently impacted by a semester-long, once-weekly class [20].

Of the 14-HST, recognizing the impact of time delays on cause-and-effect relationships (H13) had the most significant improvement, indicating that various outcomes can arise depending on the time elapsed between the cause and effect. Identifying leverage actions (H10) and seeking the most effective solutions based on the underlying system using an understanding of the system structure improved significantly. Considering mental models’ effect on the present and future (H9), considering both short- and long-term consequences (H11), and understanding the big picture (H1) also improved considerably. This suggests that the students were trained to examine phenomena from a broader perspective and consider the underlying systems. In contrast, identifying the circular nature of complex cause-and-effect relationships (H4) improved the least. Understanding whether the results can occur cyclically is a relatively challenging concept [21].

These findings help explain why the “Systems Thinking” subscale showed significant growth, since items such as H1 (understanding the big picture), H9 (recognizing the effect of mental models), H10 (identifying leverage points), H11 (considering long- and short-term consequences), H13 (time delays in cause–effect), and H14 (recognizing multiple perspectives over time) are conceptually embedded in systems thinking theory [22, 23]. Similarly, the increase in “Team Learning” is consistent with Senge’s five-disciplines model [24], because students applied these systems thinking skills in collaborative class settings, reinforcing shared understanding and practical problem-solving [25]. The improvement in the “Total” score reflects the integrated nature of these disciplines, highlighting how gains in systems thinking and team learning contribute to the broader development of a learning organization [22, 23].

The group activities showed that the students were interested in various HSS domains and had experienced and considered them. Specifically, four domains were highlighted: (1) healthcare structure and processes, (2) healthcare policy and economics, (3) clinical informatics and health technologies, and (4) healthcare value. First, in healthcare structure and processes, difficulties in healthcare delivery systems focused on where referrals from KM clinics to higher-level medical institutions tended to be seen as treatment failure rather than continuity of care. Moreover, there are no tertiary medical institutions in the KM clinics. It is essential to consider the role of KM clinics within the healthcare delivery system and its benefit to society, involving input from patients, healthcare providers, medical educators, and policymakers. Second, regarding policy and economics, the diseases included in the collaborative care pilot project were based on previous evaluations of the effectiveness of collaborative care. The ‘Senior fixed-rate system’ aimed to reduce the burden of medical expenses for the elderly but it has led to a reluctance among seniors to seek treatments that exceed the fixed rate. Third, regarding clinical informatics and health technologies, in-depth investigation topics frequently involved medical informatics and technology, likely reflecting third-year students’ efforts to understand KM using their existing scientific knowledge. Early clinical exposure appears to connect prior and subsequent learning effectively. A notable frustration was the limited public knowledge of scientific advancements in KM. In addition, the students were familiar with utilizing information technology as a clinical tool, such as using AI chatbots for clinic promotion or setting effective keywords for patient searches. Fourth, regarding the healthcare value, a notable observation was the application of disposable cups in cupping therapy in Korea, an advanced form of infection prevention. Korean cupping devices, which allow easy and minimal pain removal, have been standardized to minimize patient discomfort and risk of injury. In summary, the students showed significant interest in patient-family-community dynamics, structure and process, informatics, and technology. There has been relatively less focus on system improvements. Most of all, the students had opportunities to think about the relationships between diverse domains and understand that many factors other than the ‘disease itself’ are involved in patient’s health care. In HSS, it is essential to recognize each system and understand their interconnections and cycles [26]. Systems thinking is an essential competency for future KMDs and is expected to be the basis for learning in various fields in the future [27, 28].

The qualitative findings from the group activities, reflecting students’ interests and experiences across these four highlighted HSS domains, were triangulated with quantitative evaluations provided by clinical instructors. Whereas students most frequently highlighted domains such as healthcare structure and processes, policy and economics, informatics and technology, and healthcare value, instructors’ ratings more strongly emphasized professionalism and patient–family–community dynamics. This partial mismatch indicates that students tended to emphasize novel or conceptually stimulating domains, while instructors focused on competencies that manifested in observable clinical behaviors. For instance, although students often discussed healthcare delivery system challenges, instructors were more cautious in rating their demonstrated systems thinking. Similarly, enthusiasm toward informatics and technology was evident in reflections, but less so in instructors’ assessments of practical application. Aligning these perspectives underscores the complementary value of methodological triangulation: students’ voices capture aspirations and emerging awareness, while instructors’ ratings benchmark performance in practice. Together, these findings highlight the need for curricula that bridge reflective insight with observable competency development across all seven HSS domains.

Based on the program evaluation performed by students and clinical supervisors, the program needed more system improvement education, as students did not experience problem solving. In external medical institutions, providing problem-solving experiences can be beneficial, although challenging. Exploring such activities in university-affiliated KM hospitals or simulating problem-solving activities through project-based learning may be feasible. In addition, issues have been raised regarding the differences in medical education between university-affiliated hospitals and primary care facilities. Notably, most KM practitioners work in primary care facilities without specialist training, and university hospitals are secondary medical institutions. Therefore, the ECE program focused on primary care institutions that students found more relevant and interesting because of similarities in their future careers.

This course teaches HSS using ECE so that students’ observations and experiences can provide a clear and structured understanding of HSS realistically. The ECE was favored by students in this course. Younger students find it challenging to appreciate the connection between humanities and social medicine and clinical medicine, resulting in lower motivation for these subjects [29–31]. This study revealed that ECE effectively demonstrates the relevance of these areas. In addition, observing the daily activities of clinic directors provides insights into KM practitioners’ roles in patient-staff interactions. Interviews with practitioners highlighted lifelong learning through weekend conferences and study sessions, emphasizing continuous self-improvement. Group presentations enabled students to learn about different clinical practices, aiding in career exploration by understanding the strengths of diverse characteristics, including sex, age, and practitioners’ previous careers. Especially, the improvement in systems thinking scores observed throughout the study can be explained by the structured curricular scaffolding described in Fig. 1, which incrementally builds foundational knowledge and promotes integration of complex concepts. The summative small group discussions in particular may have solidified students’ systems thinking skills, as indicated by the notable increase in Team Learning scores. Moreover, this study provided preliminary support for the applicability of the systems thinking assessment tool within a medical education setting, demonstrating its feasibility and relevance for measuring health systems thinking competencies among medical students. Through the combination of experiential learning, scaffolded curriculum design, and validated assessment tools, this approach effectively fosters and evaluates systems thinking in health professions education.

However, this study had some limitations. Competencies in medical humanities and social medicine should be continuously cultivated and not concluded after a single semester of learning. This study reports on the effects of a one-semester course. However, it does not evaluate whether student interest, specifically their awareness of the importance of humanities and social sciences, persists during the study of clinical subjects and beyond. Future studies could examine the perceived importance of the HSS core domains specialized fields. In addition, respondent bias including social desirability bias may have influenced the self-reported data. Participants might have provided responses they perceived as more socially acceptable rather than their true beliefs or experiences. This potential bias should be considered when interpreting the results. Traditionally, students have viewed subjects such as statistics, humanities, and policy as unrelated to their major compared to direct disease treatment. While presentation outcomes showed increased student interest and recognition of these subjects’ relevance to patient care and future medical practice, quantifying these changes would be beneficial.

## Conclusions

ECE is an effective method for teaching HSS. A semester-long lecture and practicum on HSS can broaden students’ perspectives on phenomena and cultivate a long-term vision among them. Additional research on the duration and education methods is necessary to ensure sufficient learning of unique concepts, such as system improvement and the understanding of cyclic structures within systems. Moreover, diverse approaches and studies are required to explore methods for introducing HSS.

## Supplementary Information

Supplementary Material 1. Supplementary file 1. The scale of systems thinking. Supplementary file 2. Self-assessment of the habits of systems thinker. Supplementary file 3. Thematic analysis of students’ reports.

## Data Availability

All data are available from the corresponding author upon reasonable request.

## Abbreviations

AI: Artificial Intelligence
EMR: Electronic Medical Record
HSS: Health Systems Sciences
KM: Korean Medicine
LMS: Learning Management System
