# Supplementary material for: Effect of early clinical exposure based health systems science course in the Korean medical education: a prospective observational study
Source: BMC Med Educ. 2025 Dec 26;25:1713. doi: 10.1186/s12909-025-08250-z (PMC12742189; doi:10.1186/s12909-025-08250-z)
Supplement: Supplementary file 1 — Supplementary Material 1. Supplementary file 1. The scale of systems thinking. Supplementary file 2. Self-assessment of the habits of systems thinker. Supplementary file 3. Thematic analysis of students’ reports. [file 12909_2025_8250_MOESM1_ESM.docx]

Supplementary file 1. The scale of systems thinking

| Questions | | 5 | 4 | 3 | 2 | 1 |
| --- | --- | --- | --- | --- | --- | --- |
| MM | I try to view news articles or TV/internet news with a critical perspective | □ | □ | □ | □ | □ |
|  | If I do not achieve the desired results, I always take time to reflect. | □ | □ | □ | □ | □ |
|  | I spend time at least once a week thinking about who I am. | □ | □ | □ | □ | □ |
|  | I consider both the overall structure of the group I belong to and the parts that make it up. | □ | □ | □ | □ | □ |
| IS | I always consider the current situation when making plans | □ | □ | □ | □ | □ |
|  | When setting goals, I always consider how achieving them will affect me | □ | □ | □ | □ | □ |
|  | The subjects I study have a significant impact on my future and career decisions | □ | □ | □ | □ | □ |
|  | I consider how my actions will affect the future. | □ | □ | □ | □ | □ |
| TA | I actively participate in group learning activities | □ | □ | □ | □ | □ |
|  | I actively share my opinions during discussions | □ | □ | □ | □ | □ |
|  | I often take on the role of team leader in group activities | □ | □ | □ | □ | □ |
|  | I prefer group-based learning (collaboration, discussions, debates) over lecture-based learning. | □ | □ | □ | □ | □ |
| ST | When faced with a problem, I consider multiple solutions | □ | □ | □ | □ | □ |
|  | When confronted with difficult situations, I always consider how these situations arose | □ | □ | □ | □ | □ |
|  | When given different situations, I am good at finding commonalities between them. | □ | □ | □ | □ | □ |
|  | When problems arise, I try to understand the situation from different perspectives. | □ | □ | □ | □ | □ |
| SV | I am receptive to the opinions of other group members during group activities | □ | □ | □ | □ | □ |
|  | I always think positively about the outcomes of given problems. | □ | □ | □ | □ | □ |
|  | I always listen to others’ opinions. | □ | □ | □ | □ | □ |
|  | When making decisions, I consider the opinions of others. | □ | □ | □ | □ | □ |

MM, mental model; IS, individual skill; TA, team activities; ST, systems thinking; SV, shared vision

5, very good; 4, good; 3, neutral; 2, poor; 1, very poor

This questionnaire is based on one reported in another study, Lee H, Kwon H, Park K, Lee H: An instrument development and validation for measuring high school students' systems thinking. *Journal of the Korean association for science education* 2013, 33(5):995-1006.

Supplementary file 2. Self-assessment of the habits of systems thinker

Please evaluate your habit of systems thinking on a 5-point Likert scale, selecting one of the following: Very Good - Good - Neutral - Poor - Very Poor.

| Habits of a systems thinker | 5 | 4 | 3 | 2 | 1 |
| --- | --- | --- | --- | --- | --- |
| Seeks to understand the big picture | □ | □ | □ | □ | □ |
| Observes how elements within systems change over time, generating patterns and trends | □ | □ | □ | □ | □ |
| Recognizes that a system’s structure generates its behavior | □ | □ | □ | □ | □ |
| Identifies the circular nature of complex cause-and-effect relationships | □ | □ | □ | □ | □ |
| Makes a meaningful connection within and between systems | □ | □ | □ | □ | □ |
| Changes perspectives to increase understanding | □ | □ | □ | □ | □ |
| Surfaces and tests assumptions | □ | □ | □ | □ | □ |
| Considers an issue fully and resists the urge to come to a quick conclusion | □ | □ | □ | □ | □ |
| Considers how mental models affect current reality and the future | □ | □ | □ | □ | □ |
| Uses understanding of system structure to identify possible leverage actions | □ | □ | □ | □ | □ |
| Considers short-term, long-term and unintended consequences of actions | □ | □ | □ | □ | □ |
| Pays attention to accumulations and their rates of changes | □ | □ | □ | □ | □ |
| Recognizes the impact of time delays when exploring cause and effect relationships | □ | □ | □ | □ | □ |
| Checks results, and changes actions if needed: successive approximation | □ | □ | □ | □ | □ |

5, very good; 4, good; 3, neutral; 2, poor; 1, very poor

This questionnaire was adapted into a self-assessment format based on what was reported by other researchers: Skochelak SE, Hammoud MM, Lommis KD, Borkan JM, Gonzalo JD, Lawson LE, Starr SR: AMA Education Sonsortium Health Systems Science, Second edn. Philadelphia: Elsevier; 2021.

Supplementary file 3. Thematic analysis of students’ reports

| Theme  (HSS Core Domain) | Subtheme | Quotation / Description | Report Type |
| --- | --- | --- | --- |
| 1. Patient, Family, and Community | Patient experiences and beliefs | "A former guardian visiting same medical center as patient"; "Patients choose centers based on recommendations from close acquaintances." | ECE, In-depth |
|  | Patient loyalty factors | "Patients feeling genuinely cared for and receiving kind treatment." | ECE |
|  | Expectations of medicine and lifestyle | "Patients' recognition and expectations of conventional medicine, Korean Medicine (KM), and lifestyle management." | ECE |
|  | Korean medical management of HIVD patients | "HIVD management based on occupation, environment, and past medical experiences." | In-depth |
|  | Patient loyalty and communication | "Factors contributing to loyalty to a KM clinic" and "traditional knowledge explanation such as pulse diagnosis." | In-depth |
| 2. Health Care Structure and Process | Clinic consultation process | Attention to "patient reception," "waiting," "consultation," and "treatment" processes in clinics. | ECE |
|  | Professional collaboration | Observed teamwork and coordination among diverse healthcare staff. | ECE |
|  | Healthcare delivery system challenges | Referrals from KM clinics to higher-level institutions viewed as treatment failure rather than care continuity. | ECE |
|  | Differences between clinics and hospitals | Institutional differences, patient groups, inpatient numbers, required staff, and cost differences between KM clinics and hospitals. | In-depth |
| 3. Health Care Policy and Economics | Government policies impacting healthcare utilizations | Policies such as "Elderly Fixed Outpatient Copayment," and pilot projects involving collaborative treatments and herbal medicine coverage. | ECE |
|  | Payment and compensation systems | "Automobile insurance," "industrial accident compensation," and "medical aid" influencing healthcare utilization patterns. | ECE |
|  | Collaborative treatment projects | Examination of 'collaborative treatment' pilot projects between Western and Korean Medicine. | In-depth |
|  | Insurance and moral hazard | Changes in indemnity insurance inclusion of KM treatments and moral hazard issues for patients and doctors. | In-depth |
|  | Pharmacopuncture insurance | Coverage conditions and significance of insurance for pharmacopuncture treatments. | In-depth |
| 4. Clinical Informatics and Health Technology | Use of Electronic Medical Records (EMRs) and handwritten charts | Students learned practical handling of Electronic Medical Records and observed claim submission processes. | ECE |
|  | Application of research and clinical guidelines | Use of clinical guidelines, research results, and presentation of clinical outcomes. | ECE |
|  | Use of AI and technology | Use of AI chatbots for clinic promotion and customized keywords for patient searches. | ECE |
|  | Therapeutic mechanisms of KM treatments | Mechanism of cupping therapy and pharmacopuncture components, effects, and side effects. | In-depth |
|  | Standardization and clinical guidelines | Development and use of clinical guidelines and scientific standardization of KM treatments. | In-depth |
|  | Science and technology applications | Exploration of AI chatbot consultations and related customized clinical tools. | In-depth |
| 5. Population, Public, and Social Determinants of Health | Demographics and health disparities | Characterization of population by socioeconomic status, occupation, and health status differences. | ECE |
|  | Tailored lifestyle guidance | Lifestyle advice adjusted for patient's occupation and environment. | ECE |
|  | Clinic location and patient demographics | Study of KM clinic locations and major treatment subjects. | In-depth |
| 6. Value in Healthcare | Safety and infection prevention | Clinic safety measures including waste disposal, safe needle removal, infection control, and use of disposable cupping cups. | ECE |
|  | International standards in KM | Designation of a Korean cupping device as an international standard emphasizing patient safety and infection prevention. | In-depth |
| 7. Health System Improvement | Continuous learning and improvement initiatives | Clinic director attending conferences and implementation of privacy protection and accident prevention measures. | ECE |
